# The economic burden of malaria on the household in south-central Vietnam

**DOI:** 10.1186/1475-2875-7-166

**Published:** 2008-08-27

**Authors:** Chantal M Morel, Ngo Duc Thang, Nguyen Xuan Xa, Le Xuan Hung, Le Khan Thuan, Pham Van Ky, Annette Erhart, Anne J Mills, Umberto D'Alessandro

**Affiliations:** 1 LSE Health, London School of Economics, Houghton Street, London, UK; 2 National Institute for Malariology, Parasitology and Entomology, Luong The Vinh street, BC 10200 Tu Liem District, Hanoi, Vietnam; 3 Provincial Centre for Malariology, Parasitology and Entomology, 156 Ngo Gia Tu Street, Phan Rang city, Ninh Thuan Province, Vietnam; 4 Prince Leopold Institute of Tropical Medicine, Nationalestraat 155, 2000 Antwerp, Belgium; 5 Department of Public Health & Policy, London School of Hygiene & Tropical Medicine, Keppel Street, London, UK

## Abstract

**Background:**

Each year, several thousand cases of malaria occur in south-central Vietnam. Evidence from elsewhere suggests that malaria can have an economic impact on the household as the illness prevents households from completing their normal, physically demanding, productive duties such as tending crops and animals. The economic impact of malaria on households was explored within the Raglay ethnic minority living in the montainous and forested area of south-central Vietnam (Ninh Thuan Province).

**Methods:**

Two-hundred fifty-one malaria patients were identified and interviewed in an exit survey at Community Health Centres. The same patient sample was then re-interviewed in a household survey two to four weeks later. Survey data were complemented by approximately 40 informal discussions with health workers, vendors, patients, and community leaders.

**Results:**

Each episode of malaria was estimated to cost the patient's household an average of 11.79 USD (2005 prices), direct costs for travel and treatment representing 6% of the total while the remainder was loss in annual income.

**Conclusion:**

Whilst government provision of malaria treatment keeps the direct costs relatively low, the overall loss in income due to illness can still be significant given the poverty amongst this population, especially when multiple cases of malaria occur annually within the same household.

## Background

Thousands of cases of malaria occur each year in the mountainous and forested provinces of south-central Vietnam [1,2]. Whilst few of these cases are fatal, the ensuing morbidity may have an economic impact on the population. The burden of malaria can be felt both in terms of the direct costs of seeking treatment as well as the indirect costs of reduced household productivity [3-6]. Between 2004 and 2006, the National Institute of Malariology, Parasitology and Entomology of Vietnam, in collaboration with the Institute of Tropical Medicine in Belgium and with the financial support of the Belgium Cooperation and the UBS Optimus Foundation, piloted a cluster-based randomized trial to evaluate the efficacy of long-lasting insecticidal hammocks (LLIH) for the control of forest malaria in the province of Ninh Thuan. Ten intervention clusters received free LLIHs and were followed over two years, together with 10 control clusters (total 20,000 inhabitants), using bi-annual malariometric surveys and passive case detection. The latter was carried out at Commune Health Centres (CHC) and at the homes of village health workers (VHW) who were trained in the use of rapid diagnostic tests and malaria treatment. An ancillary health economic study was set up to evaluate the cost-effectiveness of this new intervention. This paper presents an estimation of the economic costs of a malaria episode to households in this area. The full economic cost and relative cost-effectiveness of LLIHs will be addressed in a subsequent paper.

## Methods

### Study area and population

The study was carried out in two districts (Bac Ai and Ninh Son) in the northwest of Ninh Thuan Province. The area is inhabited primarily by the Raglay ethnic minority, a largely self-sustaining farming community. Malaria transmission in this area is perennial with two peaks, at the beginning and end of the rainy season (May and November) [7]. Over the past few decades, the Vietnamese government has tried to relocate the Raglay community away from the forests and into purpose-built villages containing basic infrastructure. However, Raglays, a traditionally nomadic minority, continue to frequent the forest for cultivation and various other daily activities, and thus are continuously exposed to forest-dwelling mosquitoes.

Ill patients have the option of seeking care from the VHW who can do a rapid diagnostic test (Paramax 3^®^, Zephyr Biomedicals, India) and, if positive, treat patients or refer them to the CHC. Alternatively, patients can go for treatment directly to the CHC where antimalarial drugs are provided free by the government due to the poverty in the area. In another study, the treatment seeking patterns for malaria within this population were estimated to be 60% at the VHW, 38% at the CHC and 2% at other facilities [8]. Severe malaria cases are referred to the district or provincial hospital, where care is also free to the patient. The study area covers 30 villages (each with a VHW working from home) which make up 10 official communes (each with a CHC), two District Health Centres (DHC), and one district hospital.

### Methodology

Data on direct and indirect costs of malaria to the household were collected by health workers using an exit survey and a household survey. The surveys were piloted for two months and then carried out between October 2004 and March 2006. Supervision for both surveys was provided by members of the provincial and national Malaria Control Programme, who also conducted refresher sessions regarding quality of responses and met regularly with data collectors to ensure that study guidelines were adequately followed.

Patients for the exit survey were identified at the CHC after having tested positive for malaria (using a rapid diagnostic test that detects all four human malaria species). Only patients with a positive test were asked to take part. Patients were asked about all direct costs they incurred, including all expenditures associated with seeking treatment as well as all non-medical costs such as transport costs. Household visits took place two to four weeks after the initial interview at the CHC. The household survey included further questions regarding cost of any initial or follow-up treatment for malaria, productive activities, household assets, and workdays missed due to malaria. All patients included in the study were given an individual code number to maintain confidentiality.

Direct costs were calculated per patient per facility and then complemented by actual (non-trial) local treatment-seeking probabilities at each level in order to estimate the probability-weighted mean direct cost per episode within the community. The availability of free government-sponsored malaria treatment was confirmed both in the survey and through informal discussions with patients, providers and provincial health staff.

Indirect costs refer to the productivity lost within the household due to illness. Following the broad interpretation of the human capital approach [9], these costs include: reduced paid production due to the individual's disease, reduced unpaid production due to the individual's disease, and indirect costs accrued by the family for taking care of the individual during their illness. The productivity loss within the household translates to an income loss that can be calculated using various microeconomic tools. Output-based approaches value the product forgone within the loss of time until the person is replaced or recovers [10]. They attach zero value to unpaid housework (primarily due to the difficulty of making accurate estimates of its output). Opportunity cost approaches use average wages paid to local workers as proxies for the value of work of unpaid workers[9]. As the main profession in the study area was subsistence farming, formally-paid wages were not available to act as proxies for unpaid work. An output-based approach was therefore chosen to measure productivity loss due to malaria. In using this approach, income and production were taken to be expressions of the same quantity: the value-added of productive persons or their contribution to overall wealth [11]. All agricultural work was considered to contribute to income whether the products were eventually sold or consumed within the household.

It was judged that questions concerning loss of productivity due to illness would not attract accurate responses. Therefore, the household survey collected data on yearly household production (of livestock and agricultural produce) which was valued using the price of each item on the local market and taken to be a proxy for yearly income. Responses about local prices of agricultural produce and livestock varied little (average 22% deviation from the mean) so they were considered sufficiently accurate. These values were then complemented by evidence on productive days per year, work time, division of work within the family, and likelihood of being replaced when ill, in order to calculate the average daily household income per person.

Anthropological investigation associated with the study previously found that men and women work the same hours and with the same intensity in the fields and that children over the age of 10 are fully contributing members of the household. Their contribution to output was therefore given equal value. This investigation also found that this community works approximately six days per week throughout the rainy season, which corresponds to approximately half the year. Assuming that these families work only four days per week during the dry season when there is less to cultivate, the average number of household productive workdays per year is 261. Workday equivalents for working time lost were calculated assuming responses expressed in days equalled one workday and responses expressed in hours and minutes equalled a fraction of a 12-hour workday. Given the stated average number of workdays lost for the patient and carer, the average income loss per malaria episode could then be calculated per household.

Indirect and direct costs were combined to estimate the total cost incurred by the household per episode. Costs were converted from Vietnamese Dong (VND) to US dollars (USD) using the average inter-bank exchange rate for the period in which the study was conducted (15,989 VND per USD) [12]. Data entry, cleaning, and analysis were done with Epi-Info6 and Microsoft Excel. Sensitivity analysis was carried out to account for uncertainty surrounding some of the data and to test results given differing conditions and assumptions. Individual data values considered uncertain were replaced with alternate values in order to observe the level of change in the study results.

## Results

### Characteristics of study respondents

For the household survey, 251 patients were interviewed, with a sex ratio male to female of 1.67. The median age was 16, ranging from 1 to 68 years old and was similar among men (16.5 [range 1–68] and women (15 [range 1–60]). Seventy-five patients (30%) were children under the age of 10 who are considered here to be too young to contribute significantly to household production. This proportion was the same as in the general population [7] and slightly lower than the symptomatic population of which under 10s made up 36% of the population. Ninety-four percent of households sold agricultural products, 89% sold animals, and 3% worked for the public authority. Thirteen percent claimed to also do other unspecified work. Salaried workers had an annual average salary of 5,232,000 VND or 327 USD (range 63 to 438 USD). Seventy-two percent of patients had a radio in their household, 58% a television, 80% a bicycle, and 36% a motorbike.

### Direct costs of a malaria episode to the household

Direct costs associated with seeking treatment are reported in Table 1. Forty percent of the patients (n = 99) attended the CHC by bicycle, another 33% by motorbike (n = 84), and 27% (n = 67) on foot. Overall, patients incurred an average travel cost of 1,750 VND or 0.11 USD when seeking care at the commune level. Eight (3.2%) patients visited the CHC twice, but only four were treated for malaria during the first visit. Four (1.6%) patients in the study initially sought care from the VHW (prior to going to the CHC), paying a mean of 15,500 VND or 0.97 USD for treatment (range 0.38 to 1.63). It was not determined whether treatment costs were for consultation or drugs other than antimalarials. The mean travel cost for those visiting the VHW was 1,250 VND or 0.08 USD. Eight (3.2%) patients sought further treatment after attending the CHC: four went to hospital, three went to a private health clinic (possibly meaning a traditional healer) and one went to a pharmacy/drug seller. They paid an average of 3,000 VND or 0.19 USD for treatment with an average travel cost of 15,312 VND or 0.96 USD. If these direct costs are applied to the actual treatment-seeking patterns for malaria in the community, where 60% of visits take place at the VHW, 38% at the CHC and 2% at other facilities[8], patients can be estimated to incur a direct cost of 0.69 USD (Table 1).

**Table 1:** Mean direct costs (USD) per malaria episode

	Travel*	Treatment*	Proportion of actual (non-trial) visits**
Health facility (% of trial population attendance)			
Village Health Worker (1.6%)	0.08	0.97	60%
Community Health Centre (100%)	0.11	0.00	38%
Other treatment facility – hospital, private clinic, dispensary (3.2%)	0.96	0.19	2%
Mean total direct costs per episode under non-trial conditions			0.69 USD

*Data from survey of patients attending CHC

** Data from large passive case detection study [8]

### Indirect costs of a malaria episode to the household

Time loss due to treatment seeking. Average travel time was 14 minutes to the home of the VHW, 25 minutes to the CHC, and 38 minutes to other facilities (hospital, private clinic, pharmacy/drug seller). Average time spent at the health facility was 25 minutes at the VHW's home, 2 hours and 52 minutes at the CHC, and 1.77 workdays at other facilities. It was assumed that time lost from seeking treatment was included in the patient's response for the total duration of illness. If time lost for treatment-seeking of the survey population is applied to the actual treatment-seeking patterns of the community, patients, on average, lose the equivalent of 0.14 workdays in seeking treatment. Details of the time loss due to seeking treatment are reported as workday equivalents in Table 2.

**Table 2:** Mean time loss (workday equivalents) from treatment-seeking per malaria episode

Health facility (% of trial population attendance)	Travel*	Treatment*	Proportion of actual (non-trial) visits **
Village Health Worker (1.6%)	0.02	0.03	60%
Community Health Centre (100%)	0.03	0.16	38%
Secondary treatment – hospital, private clinic, dispensary (3.2%)	0.05	0.77	2%
Mean workday equivalents lost per episode under non-trial conditions			0.14

*Data from survey of patients attending CHC

** Data from large passive case detection study [8]

Patients were accompanied by an average of 1.11 household members (range:1–2). The household member stayed with the patient the entire duration of the visit and therefore could not replace the patient in the field during this time.

#### Time loss due to illness

The average malaria episode was found to last approximately 5 days (range: 1–10 days) – a duration over which most patients ceased all work. For those few who did work while ill, the vast majority stated that they were not able to perform at a normal level. Almost all patients received care from another household member. The caretaker was usually from the same household as the patient and ceased to work for most of the time the patient was ill.

#### Productivity and income loss

The surveys, together with numerous informal discussions with community members, suggested farming to be the primary economic activity in the area (see Table 3 for details). Livestock reared and sold in the area included primarily chickens, cows, and pigs. Households grew a mean of 3.1 million VND or 194 USD worth of agricultural produce per year of which they sold 2.4 million VND or 152 USD worth (79%). Households reared an average of 14 million VND or 869 USD worth of livestock per year of which they sold 3.4 million VND or 210 USD (24%). Revenue from the sales could be spent on goods the household itself did not produce. Interviews with village elders suggested that the larger livestock were sold when households struggled to find cash for meeting immediate needs like seeking health care. 50% of households reared one or more cows and 3% reared one or more buffaloes. Total yearly production per household – including both what was sold and what was consumed – was estimated to be 1063 USD.

**Table 3:** Mean annual agricultural and livestock assets and sales per household

**Crop**	**Mean household production/year (kg)**	**Proportion sold/year**	**Local value (USD per kg)**	**Total value produced (USD)**	**Total value sold (USD)**
Rice	435	64%	0.13	59.09	35.21
Maize	586	86%	0.10	55.76	47.98
Cashews	102	98%	0.60	61.62	60.41
Cassava	442	40%	0.05	21.45	8.64

**Total**				**193.83**	**152.23**

**Livestock**	**Mean owned/household**	**Sold/year**	**Local value (USD per unit)**	**Total value owned (USD)**	**Total value sold (USD)**

Chicken	8.3	3.30	2.13	17.65	7.02
Cow	3.0	0.70	250.17	750.52	175.12
Buffalo	0.2	0.04	250.17	50.03	10.01
Pig	1.8	0.70	24.27	43.68	16.99
Duck	0.5	0.10	1.94	0.97	0.19
Goat	0.1	0.02	45.34	4.53	0.91
Dog	0.2	0.02	9.38	1.88	0.19

**Total**				**869.26**	**210.42**

A loss of five full household productive workdays as a result of malaria (approximately 2% of the estimated 261 total household productive days) would result in a loss of approximately 325,629 VND or 20.37 USD in income. This is 2% of the value of yearly production. However, not all productive members of the household are incapacitated during an episode of malaria. It was assumed, based on local information, that each household had an average of three fully productive person equivalents (individuals between the ages of 10 and 65). As caretakers were found to be productively inactive for the duration of illness, two productive person equivalents were considered inactive in the case of malaria in a productive person and one productive person equivalent was considered inactive in the case of a non-productive person (aged 0 through 9 or over 65) with malaria. It can be assumed that these days are productive days as malaria usually coincides with the rainy season which is also an agriculturally productive season. An episode of malaria amongst productive members of the household is therefore estimated to represent a loss of two-thirds of this income (217,086 VND or 13.58 USD) and an episode amongst non-productive members is estimated to represent a loss of one-third of this income (108,543 VND or 6.79 USD). The mean weighted by age for all symptomatic cases in the study area was 177,501 VND, or 11.10 USD.

### Sensitivity analysis

Sensitivity analysis was conducted on the duration of illness using the ranges provided in patient responses. At the low end, a one-day episode would result in a loss of 2.22 USD in revenue per age weighted episode. At the high end, a 10-day episode would result in a loss of 22.20 USD in revenue per age weighted episode.

The number of productive household members was also subject to sensitivity analysis to see how smaller or larger households might fare differently. A household with only two productive members would incur a loss of income of 16.65 USD per age weighted episode. A household with 4 productive members would incur a loss of income of 8.33 USD per age weighted episode.

A sensitivity analysis was also conducted on travel costs. If the new VHW programme were to be cancelled and the CHC were the first level of care for those who currently go to the VHW, the average cost of travel would increase by approximately 15%. However, other costs which this study did not accurately calculate would likely increase significantly (see Discussion).

This study has examined the economic burden (proportion of household income lost directly or indirectly due to illness) caused by malaria. Depending on the context, burden is thought to be catastrophic when households are forced to cut their consumption of other minimum needs, sell productive assets, accrue large debt, or become impoverished [13]. It has been suggested that this occurs when the burden exceeds 10% of annual household income [14]. In this study, the burden of one episode of malaria is estimated to be 1.04% of annual household income on average. This is calculated using number of productive days lost out of total productive days so it does not change according to household assets. However, it must be emphasized that this estimated loss in income is per episode. If there are several episodes per household per year the burden on the household could be substantially more. Previous studies carried out in the nearby province of Binh Thuan estimated the entomological inoculation rate to be approximately 1 infective bite per person per year and the incidence of all malaria infections to be 0.44 per person/year[15,16]. Therefore, if there were to be on average between 0.5 to 1 malaria episode per person per year, then a family of five with two children under 10 would incur a cost of 27.15 to 54.31 USD or 3 to 5% of annual income.

## Discussion

### Impact on the household

Direct costs per episode were estimated to be 0.69 USD, and indirect costs 11.10 USD, making a total of 11.79 USD. Thus the impact of malaria on the household was mainly in terms of the indirect costs (measured as an assumed reduction in output) imposed on an already small income. Mean annual household income was estimated to be worth 1063 USD, which is equivalent to approximately 4.25 cows on the local market. The approximate total loss of 11.79 USD per household per episode of malaria should be seen in the light of this yearly income; for a household with no large saleable assets (cows or buffalo), the absolute burden of illness would be far more significant than for those with these assets.

Findings from a different study that was conducted on data from all provinces in Vietnam suggested that a 60% average reduction in malaria nationwide was associated with a 1.8% increase in annual household consumption. This translated to a mean 12.60 USD (1998 prices or 15.10 USD in 2005 prices). It is difficult to directly compare these findings with this study's findings due to the particular epidemiological and economic conditions of the south-central part of the country, as well as the different methods used. The nationwide study included households that did not experience any episodes of malaria, which could generally suggest that the estimated loss from malaria in this study, which only included households that experienced malaria episodes, may be low. However, the direct costs in Ninh Thuan province, where this study was conducted, are highly subsidized by government and therefore not comparable to a country-wide sample. A study from Sri Lanka estimated the loss of annual household income due to malaria to be 6% using wage rates[17] compared to this study's estimate of 3 to 5% loss using productivity losses.

Informal interviews conducted in this study revealed that households in Ninh Thuan sold productive assets such as a cow when under financial strain. It is difficult to estimate how many of the sales reported in the study took place under catastrophic conditions and what proportion of household income was lost to require the selling of these assets. Nor is it known what market conditions were like, and whether sales due to the need to obtain cash were at times when market prices were depressed (which might, for example, be the case if illness requiring sale of assets was concentrated at certain times of the year). It can, however, be inferred that those with large livestock to sell had a much easier time meeting emergency cash needs in times of illness.

Productivity loss was measured in terms of annual output potentially lost from missing a given number of productive workdays due to illness. However, there may be compensating mechanisms (such as to adjustments to worker responsibilities) that allow a household to make up for the missing days of work by the patient and carer. Studies suggest that compensation mechanisms exist in most formal work environments[18]. In a family setting, the taking of responsibility for patient or carer duties may seem even more likely. However, patient responses indicated that they needed almost full-time care from another household member when they were ill and, given that the 12-hour workday in Ninh Thuan is followed by household chores, there is little slack time for taking up additional work.

Responses to the surveys gave estimates for the cost of seeking care at each level of the health system. However, they did not represent the real distribution of malaria patients amongst the levels of care as all patients in the study had to attend the CHC in order to be identified for the study. In reality it is believed that 60% of malaria patients are treated by the VHW, 38% are treated at the CHC, and 2% seek care elsewhere [8].

Ideally more data from this study would have come from the level of the VHW, but the study design was constrained by the need to identify confirmed malaria cases and by the relatively small weekly load of malaria patients. These considerations meant that patients needed to be identified at a health facility, and health workers used as data collectors. Poor education levels amongst VHW were thought likely to affect their ability to carry out lengthy surveys, so the CHC was chosen as the point at which patients were selected. VHW patients were identified only if they also attended the CHC at some point. In reality there may be differences between those who sought care at the CHC and those who sought care from the VHW, however no significant differences were detected in this study: for example, both duration of illness and time off work were similar. As the CHC was the first and only point of call for most study patients, the data presented here are less robust with respect to costs associated with VHW care.

If the pilot CHW programme is ended and the CHC returns to being the first point of access for malaria care, this study estimated that this would increase overall average travel costs by 15%. However, it is likely that, in the absence of VHWs, more patients would seek care from alternative sources such as local drug sellers or traditional healers. As seen in study responses, treatment seeking outside the public services does carry a significant direct cost. Accurate estimates of the magnitude of this cost to the household could not be made with the data from this study as they did not capture patients who lacked village-level services.

### Reliability of data

The data for the economic analysis came primarily from a survey. Responses regarding assets may have omitted some assets grown (or raised in the case of livestock) on plots cultivated in the forest. Cultivating plots in the forest may not be illegal in itself, however, much secrecy surrounds any discussion on forest activities due to the illegal cutting and removal of wood from government-owned parts of the forest. Answering survey questionnaires is likely to appear extremely formal to the study population, with answers likely to be adapted to suit the perceived desires of government. When possible, informal discussions were used to complement survey data.

There may be some economic activity that was not captured in this study. For example, it is likely that the purchase of some drugs from shops will be missed as it is illegal for drug sellers to sell antimalarial drugs. This direct cost is likely to be small in a poor area like Ninh Thuan where drugs are free to the patient in the public health services. It is also possible that informal payment may not have been reported given that it is known to be illegal. This could have a significant effect on the study results.

Malaria challenges the productivity of this population year upon year, with the effect likely to be cumulative. The level of productivity that was calculated in this study to estimate income loss was the level of normal productivity minus the productivity loss estimated to result from an episode of malaria. This level of normal productivity is likely to be lower than it would be if malaria were not affecting household members year upon year. This means that the estimated total cost of 11.79 USD per episode is effectively underestimated and, conversely, that the gains that could be reaped in preventing malaria, over time, would be greater per episode.

## Conclusion

As in other parts of the world, malaria appears to impose a non-negligible economic burden on the household. Amongst farmers of the Raglay ethnic minority in Ninh Thuan, a south-central Vietnamese province with endemic forest malaria, this burden appears to be borne primarily in the form of indirect costs associated with decreased productivity. This loss is estimated to be approximately 11.10 USD per malaria episode. Due to take-up of government provision of services, direct costs remained low (0.69 USD), making up only 6% of the total. The total cost of illness was estimated at 11.79 USD per episode, a substantial burden for the poorest households in the community or for households experiencing multiple episodes.

## Competing interests

The authors declare that they have no competing interests.

## Authors' contributions

CMM designed the surveys, survey methodology, and helped in the initial training of health workers to conduct the surveys. NDT, NXX, LXH, PVK, AE, AJM and UA all contributed to survey design. NDT, NXX, LXH, LKT, PVK helped with logistics at both national and provincial level. NDT, NXX, and PVK trained health workers and supervised survey conduction. AE and NDT provided coordination between provincial, national, and international members of the research team. AJM supervised the study.
